# Brain connectivity fingerprinting as a predictive biomarker of art therapy outcomes in Parkinson’s disease

**DOI:** 10.21203/rs.3.rs-8475840/v1

**Published:** 2026-01-29

**Authors:** Augusto Ielo, Danilo Genovese, Joan Falcó-Roget, Enrico Amico, Alessandro Di Rocco, Monica Norcini, Angelo Quartarone, Maria Felice Ghilardi, Alberto Cacciola

**Affiliations:** IRCCS Centro Neurolesi “Bonino Pulejo”; Fondazione Policlinico Universitario A. Gemelli IRCCS; Sano Centre for Computational Medicine; University of Birmingham; Donald and Barbara Zucker School of Medicine at Hofstra/Northwell, Lenox Hill Hospital; NYU Langone Health; IRCCS Centro Neurolesi “Bonino Pulejo”; CUNY School of Medicine; Humanitas University

**Keywords:** neurorehabilitation, Parkinson’s disease, functional connectivity

## Abstract

Art therapy has emerged as a complementary approach to Parkinson’s Disease (PD), as it engages motor, cognitive, and emotional functions. However, individual responses to art therapy are highly variable and predictors of therapeutic efficacy are largely unknown. We hypothesized that the response heterogeneity may be related to individual patterns of brain activity and connectivity. Here, we combine functional connectomics, brain fingerprinting, and machine learning to identify such patterns and predict art therapy outcomes in PD. We mapped functional connectomes from high-resolution functional MRI of 23 patients with PD collected before a six-week art therapy protocol. We also assessed individual connectome fingerprints, examined their spatial specificity, and conducted meta-analytic functional decoding to link network topography with functional domains. Leveraging these network fingerprints, we computed topological measures and developed predictive models to identify patients most likely to benefit from art therapy, reaching an accuracy of 0.83 and a ROC-AUC of 0.80. Our results demonstrate that brain fingerprint-informed network measures can capture interindividual variability of therapy response, offering a data-driven, personalized approach to treatment. This study provides the first evidence that functional connectome fingerprints can guide personalized treatments in PD.

## Introduction

Parkinson’s disease (PD), a progressive neurodegenerative disorder, is characterized by both motor and non-motor manifestations ^1^ that reduce the quality of life of both patients and caregivers. While conventional pharmacological and advanced therapies have transformed the management of motor dysfunctions ^2^, they cannot halt the disease progression and the deterioration of quality of life in individuals with PD. In this context, creative and expressive therapies, including music, dance, and visual arts, have emerged as promising avenues for promoting motor, cognitive, and emotional wellbeing in PD. Art therapy, in particular, has gained attention as an intervention that can simultaneously engage multiple neural systems ^3,4^. Indeed, visual art creation involves fine motor control, visuospatial processing, executive and emotional functions, all of which are commonly affected in PD. Earlier studies in PD suggest that structured art therapy can enhance mood, reduce anxiety and depressive symptoms, and foster a sense of agency and self-expression ^5^. Importantly, art therapy may engage neuroplastic mechanisms, thus contributing to functional and structural brain changes that support adaptive behavior. However, the outcome of art therapy in PD may be highly variable, with some patients experiencing profound improvements, while others showing only minimal clinical benefit ^3,5,6^. Therefore, understanding the sources of this interindividual variability is critical to optimize therapy delivery and develop personalized interventions.

Recent advances in neuroimaging, computational modeling, and machine learning provide new opportunities to explore individual variability of therapeutic response. A novel concept emerging from these developments is that of the “brain fingerprint”, a unique, reproducible pattern of neural features that characterizes the specific brain organization of a subject ^7^. Connectome-based fingerprints derived from resting-state functional neuroimaging have demonstrated remarkable within-subject stability over time, while revealing substantial between-subject differences ^8^. They capture variations in network connectivity that may underlie differences in cognitive, emotional, and motor functions ^9,10^. A few studies have shown connectome identifiability in resting-state magneto encephalography (MEG) or functional magnetic resonance imaging (fMRI) recordings of patients with PD ^11,12^. Brain fingerprinting could also be used to predict individual responses to targeted intervention, as it provides a powerful framework for linking baseline profiles to therapy outcomes. Machine learning techniques are particularly well-suited to this challenge, as they can be used to model complex, high-dimensional relationships between multivariate predictors and clinical outcomes ^13,14^. These methods can lead to personalized treatments providing in the meantime crucial information about specific network features underlying therapy outcomes.

In this context, we implemented a multi-step approach combining functional connectomics, brain fingerprinting, and predictive modeling of art therapy outcomes. First, we estimated individual functional connectomes (FCs) in patients with PD before art therapy capturing the temporal dynamics of large-scale cortical networks. We then quantified brain fingerprints by assessing within-session temporal stability and deriving metrics of identifiability to obtain a measure of the uniqueness of the single participant’s connectivity profile. Moreover, we examined the spatial specificity of these brain fingerprints to identify the functional connections most strongly contributing to individual distinctiveness and to map these patterns across cortical networks. To link neural organization to cognition, we explored the association between fingerprint topography and cognitive functions using meta-analytic mapping. Finally, we tested whether fingerprint-informed topological network measures could predict the responsiveness to art therapy, thus discriminating “responder” versus “non-responder” patients. This brain fingerprinting-informed analytical pipeline can offer a novel approach to any personalized, data-driven therapeutic intervention.

## Results

**Figure 1:**
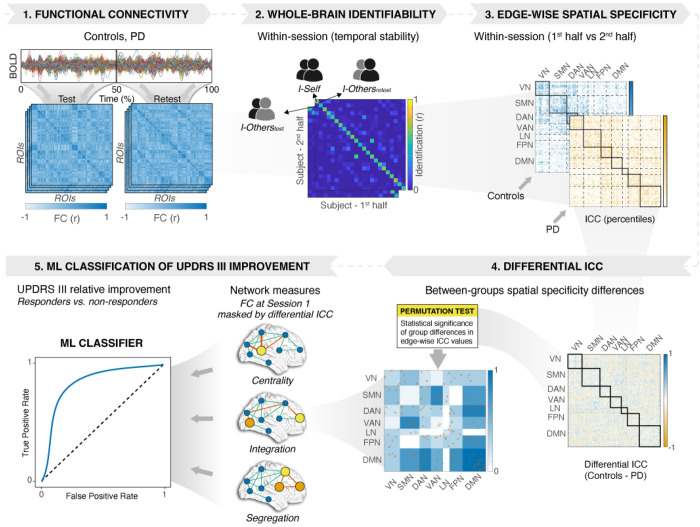
Overview of the study design and analytical workflow. 1: Resting-state fMRI data from controls and individuals with Parkinson’s disease (PD) were split into test–retest halves to compute FC matrices using the Schaefer atlas (400 regions). FC was estimated for each half-session (test and retest). 2: Whole-brain identifiability metrics (I_Self_, I_Others_) quantified within-scan temporal stability after removing confounding effects via linear regression. 3: Edgewise spatial specificity was assessed by comparing FC edges between the first and second half of each scan yielding ICC percentile maps for controls and PD. 4: Group differences in ICC values (Controls - PD) were evaluated using permutation testing to derive differential ICC matrix. 5: Network-level graph-theoretical measures (centrality, integration, segregation) extracted from FC (masked by significant differential ICC edges) were used in machine-learning models to classify PD patients as responders or non-responders based on UPDRS III improvement after art therapy.

The workflow of the present work (see Fig. 1) entailed five main steps.

1. We computed FCs for each subject, separately for the patients with PD and the control group. For each participant, we estimated Pearson correlation coefficients between the mean time series across 400 cortical regions organized into seven large-scale networks according to the Schaefer 2018 atlas ^15–17^.
2. We assessed brain fingerprinting (within-session temporal stability) by constructing identifiability matrices and computing derived metrics, including I_self_ (self-similarity), I_others_ (similarity to others), I_diff_ and I_diff–norm_ (brain discriminability), and success rate (SR) ^7,18,19^.
3. We examined the spatial specificity of fingerprints by quantifying the distinctiveness of individual FC-edges using intraclass correlation (ICC), mapping their distribution across functional networks, and projecting them onto the cortical surface.
4. We conducted meta-analytic functional decoding to link network topography with functional domains.
5. We determined whether differential ICC-derived network measures classify PD responders to art therapy.

### Demographics and Clinical Data

The PD and the control groups did not differ in age, sex distribution, years of education, or MoCA scores (Supplementary Table 1). Following art therapy, UPDRS-III scores of the PD group improved decreasing from a mean of 37.8 (SD: 11.7) to 32.5 (SD: 11.8; mean change = −5.30 [95% CI −8.39, −2.22], paired t-test: t = 3.56; p = 0.002, Cohen’s d = −0.44). Interestingly, ten patients showed a clinically meaningful improvement, defined as a ≥ 15% reduction in the UPDRS-III score ^20^. Thus, these ten patients were assigned to the “responder” group, while the other patients with < 15% improvement were classified as “non-responders”.

#### Brain connectome identifiability

**Figure 2:**
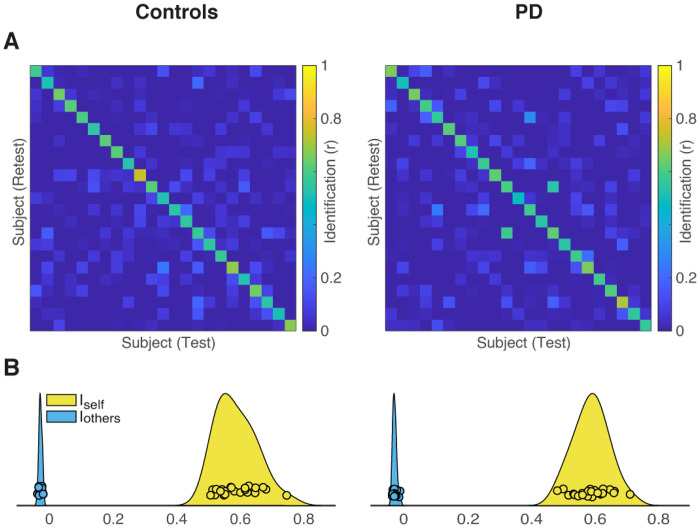
Functional connectivity-based subject identifiability in Controls and PD after residualization. Summary metrics including mean I_self_, mean Iothers, I_diff-norm_, and SR obtained after residualization are shown for each group. The distributions of I_self_ and I_others_ are summarized using kernel density estimates with overlaid individual subject values. A) Identifiability matrices for Controls and PD computed from residualized FC data. The color scale represents the identification score between test–retest FC pairs for each subject. B) Distributions of I_self_ and I_others_ after residualization. Residualization markedly reduced the between-subject similarity distribution (I_others_) to values near zero. SR always remained at 100% and an increase in differential identifiability metrics was observed.

We computed subject-level identifiability matrices from both raw and residualized FC data, and extracted I_self_, I_others_, I_diff_, I_diff–norm_, and SR in both Controls and PD patients. The results for residualized data are detailed in Table 1 and Fig. 2, whereas those for the raw data are reported in the supplemental material.

For both raw and residualized data, I_self_ (which quantifies within-subject similarity of FC) was significantly higher than I_others_ (which measures similarity between subjects) in both controls and PD (see Supplementary Fig. 1). After residualization, I_self_ remained higher than I_others_ (all p < 0.001) with SR values of 100% in both datasets. Moreover, we found that I_self_ values showed no differences between Controls and PD (Table 1). The residualization procedure markedly reduced I_others_ (Supplementary Fig. 1), without differences between the two data sets. The values of I_diff_ were 0.617 and 0.618 in Controls and PD, respectively. I_diff–norm_ was 7.23 (95% CI [3.17, 4.93]) in Controls and 6.65 (95% CI [3.05, 4.75]) in PD. Bootstrapped comparisons showed that Controls had greater I_diff–norm_ values than PD (p < 0.001). The direct comparison of I_diff–norm_ values derived from raw and residualized data with paired bootstrap procedure (1000 iterations) revealed that residualization increased I_diff–norm_ in Controls (ΔI_diff–norm_ = 2.10, 95% CI [0.02, 1.05], p = 0.036). Although I_diff–norm_ values were lower after residualization in PD, such changes failed to reach significance (ΔI_diff–norm_ = − 1.41, 95% CI [− 0.80, 0.16], p = 0.316).

In summary, we found that brain connectivity fingerprinting can be reliably applied in PD. Patients were individually identified with high accuracy based solely on within-scan connectivity patterns. Importantly, while I_self_ and I_diff–norm_ differences can be noted, PD and Control groups displayed comparable SR values, indicating that the individuality of brain connectivity profiles is preserved irrespective of clinical status. Because residualization substantially reduced between-subject similarity (I_others_) and thereby increased I_diff–norm_ values relative to estimate from raw FC (Supplementary Fig. 1), all subsequent analyses were performed on residualized data.

### Spatial specificity of brain fingerprint

We first evaluated the spatial specificity of FC fingerprints by quantifying edgewise test–retest reliability with intra-class correlation coefficients (ICCs). This metric captures the proportion of variance explained by between-subject differences relative to within-subject variability, thereby identifying FC edges that consistently reflect subject-specific patterns. ICCs were computed separately for each data set on the FC matrices estimated using the first versus the second half of the same session. To enhance ICC estimate stability, we applied a repeated random subsampling procedure, performing 100 iterations per group-condition pair, each with 80% of subjects randomly selected without replacement (see Methods). Mean edgewise ICCs from residualized FC data were moderately stable with values of 0.58 (95% CI [0.56–0.59]) for Controls and 0.58 (95% CI [0.56–0.59]) for PD.

**Figure 3:**
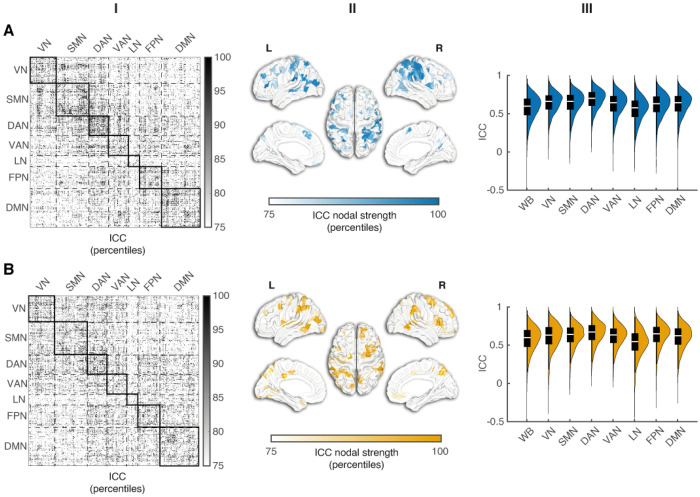
Spatial specificity of FC fingerprints in Controls (A) and PD (B). Spatial distribution and network-level patterns of within-scan ICC are shown for both groups across three complementary representations. In the first column, edgewise ICC percentile maps computed from residualized FC data. The 400×400 connectivity matrices are ordered according to the Yeo 7-network parcellation (VN: Visual; SMN: Somatomotor; DAN: Dorsal Attention; VAN: Ventral Attention; LN: Limbic; FPN: Frontoparietal; DMN: Default Mode networks) with black square delineating within- and between-network blocks and increased density points indicating higher ICC percentiles. In the second column, cortical surface maps show ICC nodal strength percentiles, defined as the sum of ICC values for all edges connected to each node. In the third column, violin plots depict ICC distributions for the whole brain (WB) and each Yeo network. Black boxes represent the interquartile range (IQR), white lines indicate the median, and whiskers denote 1.5×IQR.

We next examined the spatial distribution and network organization of fingerprint reliability for each data set. At the edge level, ICC values were broadly distributed across networks in Controls and PD (first column of Fig. 3A and 3B, respectively). In Controls, stability was greatest within the dorsal attention (DAN), somato-motor (SMN), fronto-parietal (FPN) and ventral attention (VAN) networks, with a prominent DAN-VAN between-network interaction. In PD, within-network stability was high in DAN and FPN with a pronounced contribution from the visual network (VN). Regarding between-network edges, stability was highest for DAN-FPN, VAN-FPN, and VAN-DAN. In summary, within-network stability of DAN and FPN was consistently highest in both datasets. Also, specific engagements were seen for VN in PD and for both SMN and VAN in Controls. PD showed greater involvement of visual and DAN–FPN interactions, while Controls highlighted somato-motor and ventral attention including DAN–VAN.

ICC nodal strength had distinct patterns at the network level (Fig. 3, second column) with consistent involvement of DMN and FPN. Both datasets exhibited high ICC node values (> 75th percentile) in DMN and FPN areas, displaying a very similar spatial pattern in both controls and PD participants. High ICC values were evident for DAN in both groups. Notably, in PD, additional high ICC values were found in the VN, VAN, and the limbic network (LN).

In summary, while in controls nodes with higher ICC values clustered within the DMN and the control-attention networks, in PD, reliability was concentrated in the DMN and visual networks. Therefore, the preservation in PD of DMN nodes is accompanied by increased reliability on the visual network.

Network-level ICC distributions are illustrated in the third column of Fig. 3. Rank-concordance coefficient of network profiles revealed moderate but not statistically significant differences between the two data sets (Controls vs PD: W = 0.667, p = 0.195; permutation test), suggesting that, despite lower absolute ICC values in PD, the relative ordering of networks by within-network ICC was broadly preserved across groups.

### Edgewise differential fingerprint architecture and its functional decoding

**Figure 4:**
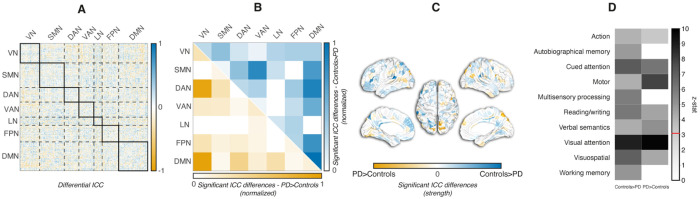
Spatial patterns of differential edgewise ICC between Controls and PD. **A.** Full differential ICC matrix (Controls - PD), with positive values (yellow scale) indicating higher reliability in Controls and negative values (blue scale) greater reliability in PD; **B**: network-level summaries of significant differences after permutation testing, with PD > Controls in the lower triangular part (yellow) and Controls > PD in the upper triangular part (blue); values are normalized to the combined total strength of significant differences; **C**: cortical maps of ICC significant group differences (blue: Controls > PD, yellow: PD > Controls). Significance was assessed using an independent-samples permutation framework (1,000 permutations). At each edge, the observed Controls-PD ICC difference was compared against an empirical null distribution derived from label-shuffled group assignments. Multiple comparisons were corected using the Benjamini-Hochberg FDR procedure, with significant edges defined at p < 0.01 (FDR-corrected); **D**: z-statistics from Neurosynth topic–based meta-analyses applied to the spatial maps of edges with significant differential ICC (Controls > PD or PD > Controls). Within each session, columns correspond to Controls > PD and PD > Controls edge sets, and rows list representative topic terms. Gray scale encodes the z-statistic (0–10); the red tick on the bar marks the significance threshold (z = 3.1).

The comparable mean levels of FC stability in both Control and PD suggest that the average temporal reliability of individual functional connections was preserved across datasets. As identifiability is also determined by edgewise ICCs spatial distribution across the connectome, we used a null model framework based on spatial permutations of edge-level ICC values (ICC_diff_) to test whether the spatial configuration of reliable connections contributed to the fingerprint structure beyond what was expected by chance. Figure 4 and Supplementary Fig. 2 illustrate the results of the comparisons in the spatial distribution of edgewise ICC values between Controls and PD, as determined by the permutation test. The full ICC_diff_ matrix (Fig. 4A) had a heterogeneous distribution of ICC differences, with both Controls > PD and PD > Controls edges. After permutation testing and FDR correction, only a limited subset of edges survived (Fig. 4B), reflecting spatially structured group differences and accounting for 64% of the total summed differential ICC across network interactions (18/28). These were not evenly distributed (see Supplementary Fig. 2): Controls showed higher reliability than PD, mainly within DMN and in DMN links to control/attention and other systems, with the following results: DMN–DMN 10.5%, DAN–DMN 9.3%, FPN–DMN 8.1%, SMN–DMN 7.5%, VAN–DMN 6.1%. By contrast, PD showed greater reliability than Controls within visual regions and their connections with DAN and DMN: VN–DMN 17.8%, VN–DAN 17.4%, VN–VN 5.4%. Cortical projections (Fig. 4C) revealed significant group differences in parietal and lateral prefrontal cortices and, to a lesser extent, in occipital and temporal cortices.

In summary, Controls exhibited significantly higher fingerprint reliability than PD across a subset of edges, yielding a spatially heterogeneous but robust pattern distributed across multiple networks, while edges predominantly in VN showed higher reliability in PD (Fig. 4A). The pattern of reduced local stability in PD likely reflects disease-relevant alterations in network organization.

Meta-analytic decoding of the significant edge sets was consistent with the network-level findings. Controls > PD edges (Fig. 4D) showed the strongest associations with cued attention, reading/writing, visuospatial functions and working memory (z > 3.1, above the significance threshold), whereas PD > Controls edges were most strongly linked to visual attention and motor function.

### Differential ICC-derived network topology measures classify responders to art therapy

From the ICC_diff_ analysis comparing Controls and PD, we identified a subset of edges with significantly higher ICC in Controls than in PD (FDR-corrected p < 0.01). This subset comprised 104 edges connecting 147 unique ROIs. We focused on these edges because they represent connectivity patterns where fingerprint stability is selectively reduced in PD and thus may capture disease-relevant alterations in network organization. By grounding our analysis to disease-related patterns, we could directly test whether reduced fingerprint reliability in these edges is predictive of individual clinical outcome of treatment.

**Figure 5:**
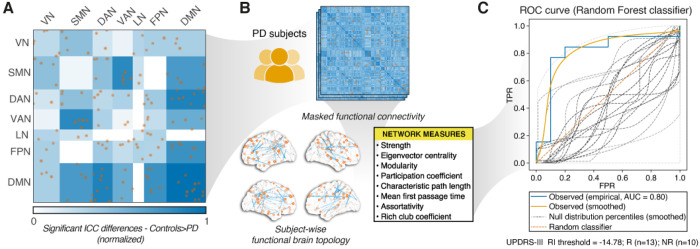
Functional network topology and classification based on ICC-masked connectivity. **A)** Matrix of significant ICC differences between controls and PD patients (Controls>PD), computed across Yeo-7 network pairs. The colormap indicates the strength (sum of weights) of edges with significantly greater identifiability in Controls, normalized within each cell. Yellow dots represent the individual ROI connections retained by the permutation-based ICC mask (FDR-corrected). **B)** For each PD subject at baseline, the FC matrix was masked using the ICC-difference map (from panel A) and a graph representation wasconstructed using only the retained edges. From this subject-wise masked graph, eight network measures were computed as reported in the inset. **C)** The resulting subject-wise dataset of graph measures was used as input to a supervised classification framework aimed at discriminating between responders (R) and non-responders (NR) to art therapy based on changes in UPDRS-III scores. Relative improvement (RI) of UPDRS-III was computed as the change from baseline to follow-up. The ROC curve of the Random Forest classifier is shown: the blue curve corresponds to the empirical ROC derived from leave-one-out cross-validation (true positive rate, TPR vs false positive rate, FPR; AUC = 0.80). The yellow curve represents the smoothed version of the observed ROC obtained via spline interpolation. Dashed grey lines indicate smoothed ROC curves corresponding to percentile levels of the null distribution generated through permutation testing, with shading proportional to the empirical frequency of the corresponding AUC values (darker grey = more frequent, lighter grey = rarer). The yellow dashed diagonal represents the performance of a random classifier.

To isolate this potentially informative subnetwork, we applied the derived ROI mask to each subject’s residualized FC matrix, retaining only the selected edges for subsequent graph-theoretical analysis. The resulting dataset of subject-wise topology measures served as input to a supervised classification framework designed to predict responder and non-responder status based on changes of UPDRS-III scores that classified 13 out of 23 participants as responders (see Methods). Importantly, by restricting the analysis to edges with reduced fingerprint stability in PD, we specifically target connectivity alterations that most likely reflect impaired functional network topology, thereby providing a feature-selection strategy for treatment response prediction. The pipeline, including ICC-based subnetwork identification, network construction, feature extraction, and classification performance (AUC), is illustrated in Fig. 5.

Among the tested classifiers, tree-based methods provided the most robust predictive performance (see Table 2). Random forest achieved the highest performance for both accuracy and ROC-AUC, followed by gradient boosting. The k-nearest neighbors classifier achieved moderate performance, outperforming the decision tree model but remaining below the best-performing tree-based approaches. Logistic regression and support vector machines showed comparatively poor results. Across all classifiers, feature importance analysis consistently identified eigenvector centrality as the most informative feature (ranked first for decision tree, random forest, and gradient boosting), supported by the recurrent contribution of assortativity and rich-club coefficient, alongside strength and mean first-passage time.

The null distributions resulting from the permutation test confirmed that model performance was significantly above chance. Specifically, for the Random Forest classifier, permutation-based one-sided p-values were p(Accuracy) = 0.004 and, p(ROC-AUC) = 0.009. After FDR correction, both Accuracy (p = 0.012) and ROC-AUC (p = 0.014) remained significant, indicating that the model reliably classified responders from non-responders beyond random expectation. To further prove our hypothesis, we also ran the classification using the PD > Controls mask. Performance markedly deteriorated: random forest (accuracy = 0.35, ROC-AUC = 0.24), gradient boosting (accuracy = 0.52, ROC-AUC = 0.56), and k-nearest neighbors (accuracy = 0.30, ROC-AUC = 0.22). These results support the specificity of the Controls > PD subnetwork for predicting clinical response.

Finally, we verified whether generic sparsification of FC provided predictive performance at least comparable to the fingerprint-informed approach. Applying proportional thresholding to residualized FC yielded lower performance than the fingerprinting-informed framework using the Controls > PD mask. Aggregated across thresholds, median (min-max) performance for the three best model families was: random forest (accuracy = 0.43 [0.30–0.56], ROC-AUC = 0.39 [0.24–0.48]); gradient boosting (accuracy = 0.39 [0.13–0.56], ROC-AUC = 0.32 [0.13–0.58]); k-nearest neighbours (accuracy = 0.48 [0.35–0.56], ROC-AUC = 0.39 [0.28–0.50]).

## Discussion

The main aim of this study was to determine whether functional connectome fingerprints derived from resting-state fMRI can predict individual responses to art therapy in patients with PD. To this aim, we first demonstrated that functional connectomes retained high levels of identifiability in both controls and patients with PD. This indicates that stable and subject-specific brain fingerprints are preserved even in the context of neurodegeneration ^12^. We then identified associations between fingerprint topography, brain networks and functional domains in the two subject groups. We found that, although mean edgewise ICCs were comparable in the two groups, edges in PD compared to controls were less stable for cued attention, reading/writing, visuospatial processing, and working memory domains and more stable for visual attention and motor functions. Finally, we showed that fingerprint-derived measures differentiated responders to art therapy from non-responders, thus providing the first evidence in PD that these measures can be good predictors of efficacy for non-pharmacological therapies.

Our finding that connectome identifiability was preserved in PD aligns with previous electrophysiological and neuroimaging reports showing stable subject-specific patterns of brain activity despite changes in network topology that may be imposed by the disease ^12,21^. The persistence of “normal” fingerprints suggests that intrinsic functional architecture remains sufficiently robust and may facilitate individualized prediction. At the same time, there is reduced reliability of certain edges in PD relative to the controls suggesting that some networks may be particularly vulnerable to the PD pathology. This conclusion is in line with prior reports of disruptions of several cortical networks^21,21,22^. Importantly, besides global reliability reductions, our approach identified local vulnerability patterns, implying that connectome fingerprinting can also delineate subject-specific network alterations.

Furthermore, we identified associations between fingerprint topography and functional domains relevant to PD. Although mean edgewise ICCs were comparable, the topography of reliability differed in the two groups. The edges had lower reliability in PD than in controls. In both groups, we found predominant connections between the DMN, the DAN/FPN, and the SMN more with smaller contributions from VN-SMN and SMN-DAN interactions (Fig. 3). The predominant connections route information between internal models, attention, and action. This is in line with prior work showing that FPN control sub-systems flexibly couple with DMN and DAN to support goal-directed cognition and task set regulation (FPN–DMN/DAN coupling), and with classic accounts of DMN’s role in internally oriented processing^23–25^.

By mapping the significant edges emerged by the differential-ICC analysis onto meta-analytic cognitive functions, we identified associations between fingerprint topography and domains relevant to PD. Edges with higher ICC in controls were most associated with cued attention, reading/writing, visuospatial processing, and working memory. Conversely, edges with higher ICC in PD were associated with visual attention and motor functions. In this framework, the predictive value of fingerprint-derived measures likely comes from capturing a pattern of selective instability at the interfaces between internal evaluation (DMN), top-down control (FPN/DAN), and sensorimotor implementation (SMN/VN). In other words, edges that are less reliable in PD provide a window onto the very circuits that art therapy exercises, thus explaining why fingerprints inform who benefits most.

Most importantly, we found that fingerprint-derived network measures could discriminate patients who responded to art therapy from those who did not. As in other studies^3,4,26^, we found improvement in UPDRS-III scores following art therapy. Furthermore, by leveraging connectome fingerprinting we were able to predict improvement in UPDRS-III scores, suggesting that subject-specific patterns of network organization not only capture stable neurobiological traits but also provide actionable biomarkers of treatment responsiveness. This is the first evidence that connectome fingerprints can serve as predictors of non-pharmacological therapy efficacy in PD. While art therapy likely engages complex neural and psychosocial domains that are only partially reflected in conventional outcome measures, the present results indicate that fingerprint-informed models can link baseline brain organization to motor gains. These results are remarkable, as art therapy does not specifically address motor performance. It mostly targets symptoms such as mood disturbances, cognitive slowing, and quality of life reduction that can be largely refractory to conventional clinical treatments^3^. Identifying neural predictors of response enables a personalized rehabilitation framework in which patients can be stratified according to their likelihood of benefit from specific interventions. Using tailored intervention strategies can increase efficiency, reduce costs, and improve outcomes.

Our framework complements emerging efforts in computational psychiatry and neurology that use machine learning to predict treatment response from multimodal data ^27,28^. While these approaches often emphasize imaging, genetics, or behavioral markers, our findings emphasize the predictive value of functional connectome fingerprints. Importantly, fingerprinting is inherently individualized, making it particularly well-suited for integration with machine learning pipelines that require stable, reproducible features. This approach aligns with the principles of precision medicine and could be readily integrated into clinical workflows as neuroimaging becomes more accessible and computational pipelines more streamlined. Finally, the incorporation of multimodal fingerprints—i.e., digital biomarkers extracted from the art-making process itself, such as stroke dynamics or compositional features—into predictive models could yield a comprehensive “neural-behavioral signature” of therapy responsiveness and could bridge neural organization and creative expression.

One of the major limitations of the present study is the relatively limited sample size. Despite this limitation, this study presented a rare opportunity to investigate a PD cohort systematically stratified for responsiveness to art therapy. While such datasets remain scarce, this work supports the use of brain fingerprints as predictors of non-pharmacological interventions in PD. Nevertheless, this result must be validated in larger, independent samples. This should be done keeping in mind that art therapy interventions may differ in terms of modality, duration, and instructional approach, and that PD motor and non-motor symptoms, progression, comorbidities, and treatments are highly variable. As outlined in the previous paragraphs, future research should also consider the integration of multimodal fingerprints to improve predictive power. Moreover, longitudinal designs may provide information about fingerprints temporal evolution. Finally, translation into clinical practice will require the development of automated, user-friendly pipelines capable of providing fast and interpretable predictive feedback to clinicians.

## Conclusions

Brain fingerprinting coupled with machine learning-based predictive modeling can provide a solid base for anticipating therapy outcomes at the individual level. By integrating neuroimaging, behavioral, art-derived readouts, and computational modeling it may be possible to move beyond a one-size-fits-all approach toward optimization of interventional strategies to enhance life quality in people with PD.

## Methods

We analyzed data from an open-label, prospective, exploratory trial investigating the effects of art therapy in PD (registered at ClinicalTrials.gov on June 7, 2017 with the identifier NCT03178786).

### Subjects

The sample included 23 patients with PD (age, mean ± SD = 68.5 ± 5.9 years; 34.8% male) who underwent fMRI recordings before art therapy. UPDRS motor scores were collected in all patients before and after the 20 art therapy sessions. fMRI was also recorded in 23 age-matched controls (age = 64.0 ± 10.5 years; 30.4% male). The diagnosis of PD was established according to published clinical criteria ^29,30^. Inclusion criteria were a) Hoehn and Yahr stage 2–3; b) Beck Depression Inventory II (BDI-II) score < 20; and c) Montreal Cognitive Assessment (MoCA) score > 22. Dopaminergic treatment regimens remained stable throughout the study. All assessments were conducted under participants’ regular treatment conditions. All controls were free of neurological or psychiatric disorders and did not receive art therapy intervention.

### Art Therapy program

The program consisted of 20 art therapy sessions, each approximately 90 minutes long, delivered twice a week over 10 consecutive weeks at the NYU Steinhardt Department of Arts and Art Professions. Participants were guided by three credentialed art therapists, resulting in a therapist-to-patient ratio of roughly 1:3. For every participant, therapists created an individualized study plan that encouraged emotional expression and artistic exploration, adapting it across sessions in response to patient feedback. Nine distinct art projects were completed, with a new project introduced every two sessions.

These projects were selected to accommodate PD-related motor limitations while stimulating visuospatial abilities that may be impaired. Participants were exposed to a wide range of media and techniques including clay modelling, canvas painting, collage, drawing, and, at a more advanced level, mural creation. All patients followed the same project sequence, although the technical complexity was adjusted to each individual’s skills and prior experience under therapist guidance. Activities were predominantly individual, with a collaborative group project concluding the final two sessions. Comprehensive methodological details of the intervention have been reported in a previous study^4^.

### Image acquisition parameters

MRI data were collected at the Center for Biomedical Imaging, NYU Langone Health, using a Siemens 3T Prisma scanner equipped with a 32-channel phased-array head coil. The imaging protocol comprised a high-resolution 3D T1-weighted magnetization-prepared rapid gradient-echo (MPRAGE) sequence (TR/TE/TI = 11.56/5.048/500 ms, flip angle = 8°, voxel size = 1 × 1 × 1 mm^3^), an axial T2-weighted sequence (TR/TE = 6000/109 ms, flip angle = 150°, voxel size = 0.6 × 0.66 × 5 mm^3^), and an eyes-closed resting-state fMRI dataset acquired with single-shot echo-planar imaging (TR/TE = 854/37 ms, flip angle = 52°, voxel size = 2 × 2 × 2 mm^3^; 400 volumes, total duration ≈ 5.7 minutes). All patients with PD were scanned in the ON therapeutic state.

#### Image processing

##### Preprocessing

Anatomical and functional MRI data were preprocessed using the fMRIPrep toolbox version 24.1.1 ^31^.

T1-weighted (T1w) images underwent intensity non-uniformity correction via the N4BiasFieldCorrection algorithm ^32^, followed by skull-stripping using a Nipype implementation of Advanced Normalization Tools (ANTs)-based brain extraction ^33^. Brain tissue segmentation was then performed with FSL’s fast ^34^ to classify gray matter (GM), white matter (WM), and cerebrospinal fluid (CSF). Finally, nonlinear spatial normalization to the MNI152NLin2009cAsym template ^35,36^ was performed using ANTs’ antsRegistration (ANTs 2.5.3).

For each participant’s fMRI, a BOLD reference volume was created for head-motion correction using FSL’s mcflirt ^37^ that generated transformation matrices and six motion parameters (three translations and three rotations). The BOLD reference was then co-registered to the corresponding T1w reference using FreeSurfer’s mri_coreg, followed by FSL’s flirt ^38^. Several confounding time series were extracted from the preprocessed BOLD data, including framewise displacement (FD), DVARS, and global signals from WM, CSF, and the whole brain. FD was computed using two formulations (Power and Jenkinson), and physiological noise components were captured via CompCor ^39^. In particular, tCompCor identified components from the top 2% most variable voxels, whereas aCompCor identified principal components from WM and CSF masks, ensuring minimal overlap with GM. All spatial transformations (head motion, distortion correction if applicable, co-registration, and normalization) were integrated into a single interpolation step using cubic B-spline resampling to minimize interpolation artifacts.

##### Functional Postprocessing

Postprocessing of fmriprep output was performed using the eXtensible Connectivity Pipeline (XCP-D) ^40^. In total, 36 nuisance regressors were selected from the preprocessing confounds, including six motion parameters, mean global signal, mean white matter signal, mean cerebrospinal fluid signal with their temporal derivatives, and quadratic expansion of six motion parameters, tissue signals, and their temporal derivatives ^41,42^. The BOLD data were despiked using AFNI’s 3dDespike with default parameters. Nuisance regressors were regressed from the BOLD data using a denoising method based on Nilearn’s approach. The time series were band-pass filtered using a second-order Butterworth filter, in order to retain signals between 0.01–0.08 Hz. The same filter was applied to the confounds. The resulting time series were then denoised using linear regression. The denoised BOLD was smoothed using Nilearn with a Gaussian kernel (FWHM = 6.0 mm). Denoised and smoothed data were parcellated into 400 cortical regions and 7 networks according to the Schaefer 2018 atlas ^15–17^. Parcellated time series were extracted using fslmeants, which computes the mean signal intensity for each atlas-defined region over time. Each time series was split into two halves, and separate Pearson correlation matrices were computed for each segment capturing whole brain functional connectivity (FC) patterns. This approach allowed for the examination of within session brain fingerprinting (i.e. functional temporal stability), yielding similar estimates to those obtained from data acquired across separate sessions (i.e., between-sessions fingerprint) ^43^.

#### Whole-brain connectivity

To assess subject-level identifiability from whole-brain functional connectomes, we applied a fingerprinting approach based on Pearson correlation across vectorized connectivity profiles. Within-session identifiability was estimated by splitting each fMRI time series into two halves (from herein, test and retest) and correlating the corresponding FC vectors ^19^. The result was a square identifiability matrix (M) containing pairwise correlations between all subjects across timepoints.

For each identifiability matrix (Controls and PD), the diagonal elements I_self_ (Eq. 1) represented the correlation between a subject’s (s) FC vector at baseline (i.e. FC_test_) and their own FC vector at follow-up (i.e. FC_retest_).


(1) $$I_{\mathrm{self}}(s)=\text{corr}\left(FC_{\mathrm{test}}(s),FC_{\mathrm{retest}}(s)\right)$$

The off-diagonal elements (I_others_) described the similarity between a given subject (s) and all other individuals in the sample (i), and were computed for each subject as the average of the correlations between their FC vector and those of all other participants (Eq. 2).


(2) $$I_{\mathrm{others}}(s)=\frac{\underset{i\neq s}{\sum }\left(\text{corr}\left(FC_{\text{test}}(s),FC_{\text{retest}}(i)\right)+\text{corr}\left(FC_{\text{retest}}(s),FC_{\text{test}}(i)\right)\right)}{2N-2}$$

A summary metric of identifiability (I_diff_) was computed as the difference between the mean I_self_ and the mean I_others_ across all subjects ^7^ (Eq. 3).


(3) $$I_{\text{diff}}=\text{mean}|\left(I_{\text{self}}(s)\right)-\text{mean}\left(I_{\text{others}}(s)\right)$$

Furthermore, to obtain a scale-free effect size that is comparable across conditions, we computed a normalized identifiability index (Idiff-norm, Eq. 4).


(4) $$I_{\text{diff-norm}}=\frac{\mu_{\text{within}}-\mu_{\text{between}}}{\sqrt{\frac{\left(n_{\text{within}}-1\right)SD_{\text{within}}^{2}+\left(n_{\text{between}}-1\right)SD_{\text{between}}^{2}}{n_{\text{within}}+n_{\text{between}}-2}}}$$

Where μ_within_ is the mean of the diagonal of the identifiability matrix, μ_between_ is the mean of all off-diagonal entries M_ij_ with i ≠ j, SD^2^_within_ and SD^2^_between_ are the corresponding sample variances, and n_within_ and n_between_ are the numbers of scores in each distribution ^18^.

Finally, the identification success rate (SR) was calculated as the percentage of subjects for whom the I_self_ value was higher than all corresponding I_others,_ reflecting correct matching between a subject’s baseline and follow-up connectivity profiles.

#### Edgewise spatial specificity estimation

To assess the spatial specificity of individual FC fingerprints, we computed edgewise intra-class correlation coefficients (ICCs), which quantify the test-retest reliability of each functional connection across time. The ICC measures the proportion of total variance attributable to between-subject differences relative to within-subject (i.e., residual) variance, thereby capturing the extent to which each FC edge consistently reflects subject-specific connectivity patterns.

Edgewise ICCs were estimated using a one-way random effects model, corresponding to ICC(1,1), which is appropriate when sources of variance, such as head motion, scanner instability, and physiological noise, are assumed to be present but are not explicitly modelled. For each edge connecting two brain regions, the ICC was calculated according to:

(5) $$\text{ICC}(1,1)=\frac{MS_{B}-MS_{W}}{MS_{B}+(k-1)MS_{W}}$$

where MS_B_ is the between-subjects mean square from a one-way ANOVA (inter-individual variability), MS_W_ is the within-subjects/residual mean square (fluctuations across repeated sessions within the same individual), and k = 2 is the number of repeated measurements (test and retest). This formulation allows the ICC to capture the proportion of variance due to inter-individual differences while accounting for fluctuations within individuals across groups ^19,44^.

ICCs were computed separately for each group (temporally split halves of the same session) using subject-level test and retest FC matrices. To improve the stability of ICC estimates and mitigate the influence of sampling variability, we applied a repeated random subsampling procedure. For each group, 100 iterations were performed, each time randomly selecting 80% of the available subjects without replacement. For each iteration, ICC was computed at the edge level using Eq. 5, and the resulting values were averaged to produce a robust estimate of edge-specific reliability.

The ICC is commonly interpreted in the literature as a measure of discriminability, reflecting the reliability with which a feature (here, a connectivity edge) distinguishes individuals across repeated measurements ^45^. Thresholds for interpreting ICC values have been proposed and widely adopted: values below 0.4 are considered to reflect poor reliability; values between 0.4 and 0.59 indicate fair reliability; values between 0.6 and 0.74 are considered good; and values above 0.75 are deemed excellent ^46^.

##### Differential ICC analysis

To investigate whether the spatial distribution of FC identifiability differs significantly between the two groups (Controls vs PD), we calculated the difference between the previously computed edgewise ICC maps (ICC_diff_). Subsequently, we performed statistical comparisons aimed to identify edges whose fingerprinting reliability (i.e., test-retest stability across individuals) differs significantly between groups. By quantifying such differences, we characterized spatially specific alterations in connectome identifiability in PD.

We tested differences in ICC between groups at each edge using a permutation-based framework (1000 iterations) that preserves the temporal structure of test-retest data adopting an independent-samples approach. At each iteration, test-retest ICC values were recomputed separately for the two permuted groups, and the resulting group-level differences were stored to form an empirical null distribution. The actual ICC difference (Controls - PD) was then compared against this null distribution at each edge, and p-values were computed as the proportion of permutations yielding absolute differences equal to or greater than the observed one.

Multiple comparisons were controlled using the False Discovery Rate (FDR) procedure with Benjamini-Hochberg correction. Edges surviving a threshold of p < 0.01 (FDR-corrected) were identified as significant. These corresponded to edges with significantly higher ICC in either Controls or PD. Significant edgewise differences were subsequently aggregated within and between canonical functional networks using the Yeo 7-network parcellation ^15–17^, computing the total ICC difference strength for each pairwise network combination.

To further interpret the functional relevance of edges showing significant ICC differences, we performed a meta-analytic functional decoding using the Neurosynth platform (https://neurosynth.org/) ^47^. Following procedures established in prior work ^19^, we conducted topic-based meta-analyses using 50 predefined terms from the Neurosynth database, capturing large-scale cognitive and functional domains. This analysis allowed us to infer the putative functional roles associated with group-specific connectome fingerprints, facilitating interpretation of the spatially distributed ICC differences in terms of underlying cognitive processes.

##### Statistical Analysis

All statistical analyses were performed using MATLAB R2023a (The MathWorks Inc., Natick, MA). Statistical significance was set at p < 0.05 unless otherwise specified. Normality of data distributions was assessed using the Shapiro-Wilk test for normality.

##### Demographics and clinical data

Demographic comparisons between PD and control groups were performed using independent-samples t-tests for continuous variables that were normally distributed and Wilcoxon signed-rank tests for non-normally distributed variables. Chi-square tests were used for categorical variables, such as sex.

Pre- and post-treatment differences within the PD group were assessed using paired t-tests for normally distributed data and Wilcoxon signed-rank tests for non-normal distributions. These tests were applied to evaluate changes in motor and non-motor clinical scores between baseline and follow-up sessions.

The relative improvement in UPDRS-III (ΔUPDRS-III) was computed as described by Eq. 6.


(6) $$\Delta \;\text{UPDRS-III}(\text{s})=\frac{{\text{UPDRS-III}}_{BL}(s)-{\text{UPDRS-III}}_{FU}(\text{s})}{{\text{UPDRS-III}}_{\text{BL}}(s)}\times 100$$

Where UPDRS-III_BL_ is the score obtained by each subject (s) at baseline, before art therapy, and UPDRS-III_FU_ is the score collected after the 20 art therapy sessions.

##### Identifiability measures

FC matrices were residualized for confounding variables prior to identifiability analysis. Specifically, subject-level FC data were adjusted via linear regression to remove the effects of age, sex, years of education, disease duration, and age at PD onset. For controls, the same residualization procedure was applied, setting disease duration and age at PD onset variables to zero. This choice was supported by the inter-individual variability observed in our PD sample. Residualized FCs were used consistently across all analyses.

Identifiability metrics, namely I_self_, I_others_, were computed for each subject. Comparisons between groups were conducted using independent-samples t-tests. If normality was not met, Wilcoxon signed-rank or Mann-Whitney U tests were used as appropriate. In addition, within-group comparisons between Iself and Iothers were performed using paired t-tests (or Wilcoxon signed-rank tests when normality was not met).

To assess I_diff-norm_, we applied a non-parametric bootstrapping approach (1000 iterations). At each iteration, subject-level I_self_ and I_others_ values were resampled with or without replacement (depending on whether comparisons were paired or independent), and I_diff-norm_ was recalculated. Differences between bootstrapped distributions were then compared across groups (e.g., Controls vs PD) using two-tailed percentile-based confidence intervals and empirical p-values. In addition, to directly test the effect of residualization, we employed a paired bootstrap procedure: for each iteration, the same resampled subject indices were applied to both raw and residualized identifiability matrices, yielding paired estimates of Idiff-norm. The difference between residualized and raw Idiff-norm (ΔIdiff-norm) was then computed, and its bootstrap distribution used to derive percentile-based confidence intervals and empirical p-values.

##### Comparisons of edgewise ICC stability

To further characterize differences in network stability, we compared mean edgewise ICC values across multiple experimental conditions. Specifically, we examined the differences between controls and PD patients. To provide a consistent measure of effect magnitude, we computed Cohen’s d offering a standardized index of effect size that complements statistical significance testing. In addition, we computed ICC values for each Yeo network by averaging the upper-triangular edges within the network’s submatrix of the ICC matrix (after ROI reordering according to network assignment). For each group, the seven Yeo networks were ranked according to their mean within-network ICC. We then quantified the similarity between these rankings across conditions using a concordance score, defined as the proportion of network pairs with the same relative order in both rankings. Statistical significance of the observed concordance was assessed via a permutation test (1000 iterations), in which the second ranking was randomly shuffled to generate a null distribution of concordance scores.

#### Network topology analysis and clinical response classification

To examine whether differences in connectome organization within the most discriminative edges could predict clinical response, we first identified the set of ROIs connected by edges showing significantly higher ICC in controls than in PD (Controls > PD) from the previously described ICC_diff_ analysis. Analyses were restricted a priori to this mask, on the rationale that connections stable in controls but selectively less stable in PD reflect disease-related change and carry prognostic information for clinical response. This ROI mask was used to filter each patient’s FC matrix, computed from the full time series, thereby retaining only the subset of connections within this discriminative network. We then computed a set of graph-theoretical network topology measures on the resulting filtered connectomes using the Brain Connectivity Toolbox (BCT) ^48^. The selected metrics were chosen to provide non-redundant coverage of three key topological domains: centrality, integration, and segregation, along with measures of network organization. Specifically, we extracted two representative measures for each domain: for centrality, strength and eigenvector centrality; for integration, characteristic path length and mean first passage time; for segregation, modularity and participation coefficient. Additionally, we quantified network organization using assortativity and the rich club coefficient.

The resulting subject-wise dataset of graph measures was used as input to a supervised classification framework discriminate between responders and non-responders to art therapy, based on changes in UPDRS-III scores. The relative improvement in UPDRS-III (ΔUPDRS-III) was computed as described by Eq. 6.


(7) $$\Delta \;\text{UPDRS-III}(\text{s})=\frac{{\text{UPDRS-III}}_{BL}(s)-{\text{UPDRS-III}}_{FU}(\text{s})}{{\text{UPDRS-III}}_{\text{BL}}(s)}\times 100$$

Where UPDRS-III_BL_ is the score obtained by each subject (s) at baseline, and UPDRS-III_FU_ is the score at follow-up. Subjects were classified as responders or non-responders according to their percentage change in UPDRS-III. A reduction of 15% was used as the conceptual boundary for clinically meaningful improvement, similarly to thresholds adopted in previous studies on non-pharmacological interventions for Parkinson’s disease ^20^. We implemented a nested leave-one-out cross-validation (LOOCV) scheme to ensure unbiased performance estimation and optimal hyperparameter selection. In each outer loop iteration, one subject was held out as the test case, and the remaining data were used for training. Within the training set, a grid search with k-fold cross-validation (k = 5) was performed to tune model-specific hyperparameters. Features were standardized to zero mean and unit variance prior to training in each fold, using parameters estimated from the training data only. We included three widely used models (random forest, gradient boosting and k- nearest neighbors). For each model, performance was assessed on the held-out subject using accuracy, area under the ROC curve (ROC-AUC), area under the precision–recall curve (PR-AUC), precision, recall, and F1 score.

To assess the statistical significance of the observed classification performance, we implemented a permutation testing procedure (1000 iterations) following the same nested LOOCV scheme. At each iteration, class labels (responder/non-responder) were randomly permuted while preserving the original feature structure, and the entire model training and evaluation pipeline was repeated. This yielded empirical null distributions for accuracy and ROC-AUC, against which the observed values were compared to derive one-sided p-values. False discovery rate (FDR) correction (Benjamini–Hochberg) was applied across the three metrics. Confusion matrices were averaged across outer folds to provide a summary of classification outcomes. To assess model explainability and identify the most informative predictors, we examined feature importance values derived from the fitted models. For the tree-based classifiers, feature importance was quantified using the mean decrease in impurity (MDI), corresponding to the average reduction in Gini impurity across all nodes where a given feature was used for splitting. The resulting feature importance values were averaged across folds, allowing us to rank network topology measures according to their predictive relevance for clinical response. The analysis was repeated using the mask comprising edges with significantly higher ICC in PD than Controls (PD > Controls) to confirm that predictive signal was specific to edges that are stable in controls but selectively less stable in PD (Controls > PD).

As a further analysis, we repeated the full pipeline without any fingerprint-derived mask by applying proportional thresholding to the baseline residualized FC matrices (threshold_proportional function, BCT). Densities were set to p∈[0.05,0.95] in 0.05 increments. Thresholding was applied to the absolute FC to construct non-negative, weighted undirected graphs. The same topological network measures were then computed, with mean first-passage time evaluated on the largest connected component. Features were fed to the supervised classification framework described above. This analysis was aimed at assessing whether generic sparsification of FC provides predictive performance comparable to the fingerprint-informed approach.

## Supplementary Material

This is a list of supplementary files associated with this preprint. Click to download.

• Ieloetal2025SupplementaryInformation.docx

## Figures and Tables

**Table 1:** Identifiability metrics. I_self_ and I_others_ are expressed as mean ± SD and compared using paired-sample (Controls, PD) or independent-sample (Controls vs PD) t-tests or non-parametric equivalents when normality was not met. I_diff–norm_ is expressed as median (95% bootstrap CI), obtained from a 1000-iteration non-parametric bootstrap resampling subject-level I_self_/I_other_s. Between-group p-values for I_diff–norm_ were computed from the bootstrap distribution of differences using percentile-based confidence bounds and empirical p-values. Significant differences are reported in bold.

	Controls	PD	Controls vs PD
I_self_	0.59 ± 0.06	0.59 ± 0.06	p = 0.931
I_others_	−0.03 ± 0.00	−0.03 ± 0.00	p = 0.913
I_diff–norm_	**7.23 (95% CI [3.17, 4.93])**	**6.65 (95% CI [3.05, 4.75])**	**p < 0.001**
SR	100	100	n.a.

**Table 2:** Classification performance metrics.

Model	Accuracy	Precision	Recall	F1-Score	ROC-AUC
Random Forest	0.83	0.85	0.85	0.85	0.80
Gradient Boosting	0.74	0.73	0.85	0.79	0.76
K-nearest neighbors	0.70	0.80	0.62	0.70	0.68

## Data Availability

The data used to support the findings of this study are available upon reasonable request. Data availability is subject to the restrictions imposed by the institutional review board on the use of human subjects’ data.
